# High Mobility Graphene on EVA/PET

**DOI:** 10.3390/nano12030331

**Published:** 2022-01-21

**Authors:** Munis Khan, Kornelia Indykiewicz, Pui Lam Tam, August Yurgens

**Affiliations:** 1 Department of Microtechnology and Nanoscience, Chalmers University of Technology, 412 96 Göteborg, Sweden; kornelia.indykiewicz@chalmers.se (K.I.); avgust.yurgens@chalmers.se (A.Y.); 2 Faculty of Electronics, Photonics and Microsystems, Wrocław University of Science and Technology, Janiszewskiego 11/17, 50-372 Wrocław, Poland; 3 Department of Industrial and Materials Science, Chalmers University of Technology, 412 96 Göteborg, Sweden; eric.tam@chalmers.se

**Keywords:** graphene, CVD, flexible substrates

## Abstract

Transparent conductive film on a plastic substrate is a critical component in low cost, flexible and lightweight optoelectronics. CVD graphene transferred from copper- to ethylene vinyl acetate (EVA)/polyethylene terephthalate (PET) foil by hot press lamination has been reported as a robust and affordable alternative to manufacture highly flexible and conductive films. Here, we demonstrate that annealing the samples at 60 ${}^{\circ }$ C under a flow of nitrogen, after wet etching of copper foil by nitric acid, significantly enhances the Hall mobility of such graphene films. Raman, Scanning electron microscopy (SEM) and X-ray photoelectron spectroscopy (XPS) were used to evaluate the morphology and chemical composition of the graphene.

## 1. Introduction

Ever since its discovery [1], graphene has emerged as one of the most promising 2D materials in the field of electronics and optoelectronics [2,3]. Highly conductive flexible and transparent graphene films pave the way for new developments in flexible electronics. Owing to its excellent mechanical properties, graphene has attracted substantial attention in the field of stretchable electronic devices. In addition, the other material properties, such as high chemical stability, wide optical absorption spectrum (300–1400 nm), excellent transparency of ∼97% and electrical sensitivity towards biochemicals, make it a promising material for displays, light harvesting devices and biosensors [3]. Chemical vapor deposition (CVD) of graphene on commercial copper (Cu) foils [4,5] provides a scalable route towards high-quality single-layer graphene. Most applications, however, require graphene to be transferred to a suitable target substrate. Polymer assisted transfer technique [6] is the most widely used approach to transfer graphene. This is usually conducted by applying a sacrificial polymer layer to support graphene, followed by etching away the copper and dissolving the polymer layer. In practice, such a process often results in unwanted contamination of the graphene surface by the polymer residues, impairing its electrical properties [7]. Recently, a direct lamination of graphene onto flexible substrates (such as polyethylene terephthalate, PET) has emerged as a robust and time efficient method of transferring graphene without the need to apply a sacrificial polymer layer [2,8,9,10,11,12]. This prevents the unwanted contamination and results in high-mobility graphene. After the lamination, copper foil is either removed by mechanical delamination or chemical etching. However, the mechanical delamination has been reported to cause significant deformation of graphene that greatly reduces its electrical conductivity [13,14]. Therefore, the chemical etching remains a preferred method in applications requiring high-mobility graphene. The chemical etching by HNO ${}_{3}$ , as well, can alter the properties of graphene via doping and adsorption of chemical species at the surface of graphene [15]. This surface modification is, however, weak and can be reversed by simple annealing at elevated temperatures [16]. Despite much work on a roll-to-roll lamination transfer of graphene to flexible substrates, a thorough investigation of annealing of graphene on such substrates and its correlation with the electrical properties of graphene is missing.

High intrinsic carrier mobility ( μ ) is often a primary requirement for fabrication of high-speed flexible transistors in the terahertz range [17]. Recently, there have been few approaches to fabricate graphene-based RF devices for plastic electronics [18,19]. For these applications, often highly conductive flexible graphene films are required. Electrodes are also an essential component of any electronic devices requiring high conductivity. The same is true for flexible devices, where integration of the mechanically less flexible metal electrodes results in poor device performance. Graphene on the other hand, due to its high electrical conductivity and mechanical stability, shows great potential for such applications [10,20]. Graphene has also been used as a transparent electrode in Organic LED’s [21]. This also requires highly conductive graphene films for superior optoelectronic properties. Therefore, graphene’s extraordinary electrical properties have not only drawn enormous research interest but have also been successfully attempted in various flexible, stretchable and conformal electronic applications. In this work, we report a significant increase in the mobility of CVD graphene transferred to EVA/PET by annealing the samples at 60 ${}^{\circ }$ C for several hours. CVD graphene on Cu is transferred to EVA/PET via the hot-press lamination and etching of metal catalyst by HNO ${}_{3}$ . Raman, XPS and SEM measurements were carried out to correlate such an increase in mobility to the structural, chemical and morphological properties of the graphene.

## 2. Materials and Methods

Graphene was grown on commercial copper foils in a cold-wall low-pressure CVD reactor (Black Magic, AIXTRON). A 25-μm thick copper foil was treated with acetic acid to remove surface oxides prior to CVD growth. The Cu foil was then mounted in a CVD chamber and the temperature inside the chamber was increased to 950 ${}^{\circ }$ C. In the CVD process, pre-diluted methane (5% CH ${}_{4}$ in argon, 120 sccm) mixed with hydrogen (60 sccm) was introduced in the chamber for graphene growth. The temperature inside the reactor was then decreased before finally removing the Cu foils with graphene from the chamber.

**Figure 1:**
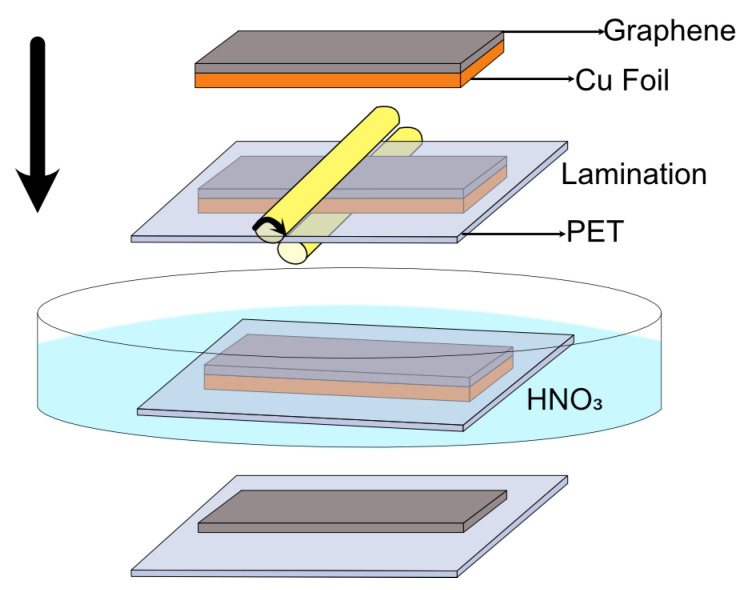
Process flow for graphene transfer from Cu foil to PET substrate.

We used hot-press lamination and chemical etching to transfer graphene grown on Cu to EVA/PET (Figure 1). Cu foils with graphene were laminated to EVA/PET using an office laminator and usual lamination pouches 125 μm thick. For removal of Cu, we used 30% HNO ${}_{3}$ . After etching Cu completely, the films were washed in deionized water followed by blow-drying with nitrogen.

Electrical characterization of ∼1 × 1 $\;{\mathrm{cm}}^{2}$ samples included the resistivity- and Hall-effect measurements using van der Pauw’s method to deduce the charge-carrier mobility and carrier concentration in graphene at room temperature and in ambient air. In particular, four-probe measurements were carried out to determine the sheet resistance ( $R_{\mathrm{sh}}$ ), while the Hall-effect measurement on the same sample were used to extract the effective carrier concentration (*n*). Given these two value, the carrier mobility μ was calculated as $\mu =1/(qR_{\mathrm{sh}}n)$ , where *q* is the electron charge. The Raman spectroscopy measurement were performed using the Horiba Raman XploRA™ microscope with a 638 nm laser. The Zeiss Supra 55VP system with 5 kV accelerating voltage was used to acquire scanning electron microscope (SEM) images of the samples. The XPS measurements were performed on PHI5000 VersaProbe III Scanning XPS Microprobe™ system with monochromatic AlK α X-ray source ( E=1486.6eV ) source. During the measurements, a dual-charge compensation utilizing an electron neutralizer and an ion gun was applied because the sample is not fully conductive. Survey scans in a range between 0 eV and 1300 eV with the energy step size 1.00 eV were conducted to evaluate the surface composition of the samples before and after annealing. Narrow scans, on the other hand, were conducted to analyze the chemical state of the selected elements, in which, the energy step size in the C1s measurements was set at 0.05 eV, whilst that was 0.10 eV in the O1s measurements.

## 3. Results and Discussion

**Figure 2:**
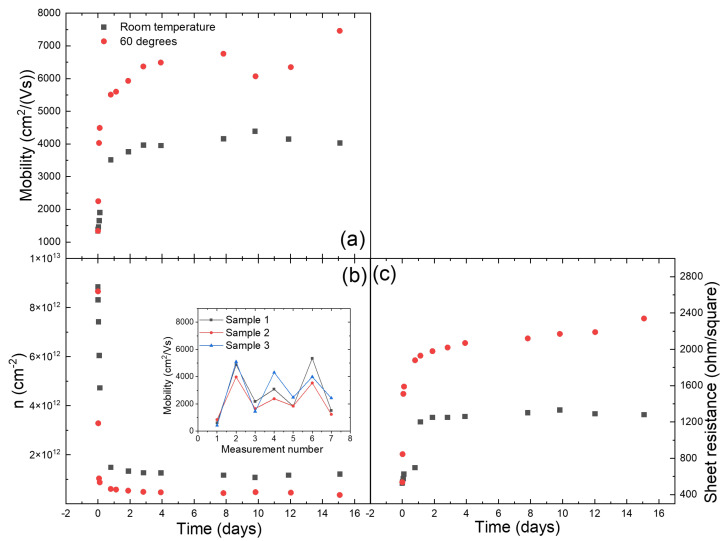
The time dependence of the (**a**) mobility μ , (**b**) carrier concentration *n* and (**c**) sheet resistance $R_{\mathrm{sh}}$ , for a few samples kept at room temperature (black squares) and at 60 ${}^{\circ }$ C in nitrogen flow (red dots). The inset: repeated HNO ${}_{3}$ treatment and annealing.

The adsorption of chemical species on the surface of graphene during its treatment with various chemicals has been known to introduce defects and dopants in graphene lattice [15], which results in increased charge-carrier scattering and lower carrier mobility [11,22]. Annealing graphene at very high temperatures is often used to remove adsorbed species. However, the annealing temperature for graphene transferred to EVA/PET is limited by the low melting point of the EVA-based glue layer of a lamination foil. For our lamination pouches, the EVA-layer starts to melt already at 70–75 ${}^{\circ }$ C. In this work, we show that despite this limitation, annealing of graphene on EVA/PET even at such a low temperate still helps to remove adsorbed chemical species. This reduces the overall doping and density of scattering centers, leading to a higher charge-carrier mobility. The carrier concentration and mobility in the samples prior and post annealing can be quickly assessed via the Hall-effect measurements. Details about the Hall-effect measurement setup and van der Pauw’s method are provided in the Supplementary Information (SI). Figure 2 shows the evolution of the mobility and charge carrier concentration with time. Two samples, one stored at room temperature and one at 60 ${}^{\circ }$ C, were measured using an in house Hall-effect measurement setup (see Supplementary Information Figure S1) for over two weeks. As seen in Figure 2, the mobility of the sample stored at 60 ${}^{\circ }$ C increases more rapidly compared to the sample stored at room temperature. We report the mobility in the range of 7000–8000 $\;{\mathrm{cm}}^{2}V^{-1}s^{-1}$ , which is a far better value than what is typically achieved after transferring CVD graphene from Cu to SiO ${}_{2}$ using the polymer assisted transfer technique (1000–2000 $\;{\mathrm{cm}}^{2}V^{-1}s^{-1}$ ) [23,24,25,26]. The increase in mobility is very fast in the beginning and eventually saturates to a stable final value. The charge-carrier concentration of the two sample is plotted in the same graph (Figure 2). The evolution of the sheet resistance of the same samples is also plotted in Figure 2. The sheet resistance, being initially low, increases to a high value for both the samples stored at 60 ${}^{\circ }$ C and room temperature. The high sheet resistance is a direct result of decreased doping of graphene and is comparable to the results reported by other groups [11,12]. As evident from the results, the charge carriers in freshly etched graphene samples have a high concentration (doping) and low mobility. This is consistent with doping of graphene transferred to EVA/PET after etching of Cu by HNO ${}_{3}$ [11], which in turn results in reduced carrier mobility. However, we observe that the carrier mobility increases with time. Such an increase in carrier mobility can be further sped up by annealing samples at 60 ${}^{\circ }$ C. As seen in Figure 2 the initial increase in mobility (and decrease in carrier concentration) is more prominent for samples stored at 60 ${}^{\circ }$ C.The sudden drop in mobility seen on the measurement recorded on day 10 can be attributed to wearing of the contacts (see Supplementary Information Figure S2 for more details). Once the contacts are revived, a similar trend of increase in mobility is followed. This can be explained by a temperature-assisted desorption of molecules from the surface of graphene. A similar observation has been reported in [16], where CVD graphene transferred to SiO ${}_{2}$ after being treated with HNO ${}_{3}$ , showed initially lower carrier mobility and higher carrier concentration. However, upon annealing such samples in vacuum, the mobility recovered to higher values. This proves that adsorption and desorption of molecules from the surface of graphene is indeed caused by its treatment with HNO ${}_{3}$ and annealing, respectively, independent of the underlying substrate. Although the annealing of CVD graphene transferred to SiO ${}_{2}$ has been studied and reported extensively, the impact of such an annealing of graphene transferred to PET is rare. We also did the control experiments to quantify the reversibility of the HNO ${}_{3}$ treatment on graphene’s electrical properties. Three samples were repeatedly treated with HNO ${}_{3}$ and annealed overnight at 60 ${}^{\circ }$ C. As seen in the inset of Figure 2b, the mobility drops every time the samples are treated with HNO ${}_{3}$ (measurement 3, 5 and 7) and increases to higher values after annealing at 60 ${}^{\circ }$ C overnight (measurements 2,4 and 6).

**Figure 3:**
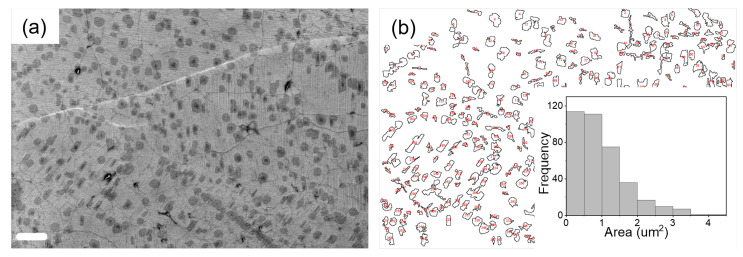
(**a**) SEM image of graphene on EVA/PET (the scale bars correspond to 4 μm). (**b**) corresponding size distribution map of multilayer graphene (dark patches in (**a**)). Inset: Histogram of area distribution of multilayer patches.

In order to check the surface morphology of our sample, SEM images were captured. Atomic force microscopy (AFM) is inherently difficult to conduct on our samples, which lack the required smoothness (see Supplementary Information Figure S4). SEM image of graphene transferred to EVA/PET reveal a large density of bilayer- and trilayer patches (the dark regions), along with wrinkles and grain boundaries as shown in Figure 3a. However, despite the presence of these defects, a high carrier mobility is recorded after annealing at 60 ${}^{\circ }$ C. In order to extract the quantitative information, regarding the multilayer coverage, the SEM image in Figure 3a was analyzed using ImageJ™ software (https://imagej.nih.gov/ij/index.html (accessed on 13 January 2022)). Figure 3b shows the distribution map of multilayer graphene patches (dark patches in Figure 3a). In the inset of Figure 3b, area-size distribution of the multilayer patches is plotted. About 17% of the area shown in Figure 3a is covered with multilayer graphene patches.

**Figure 4:**
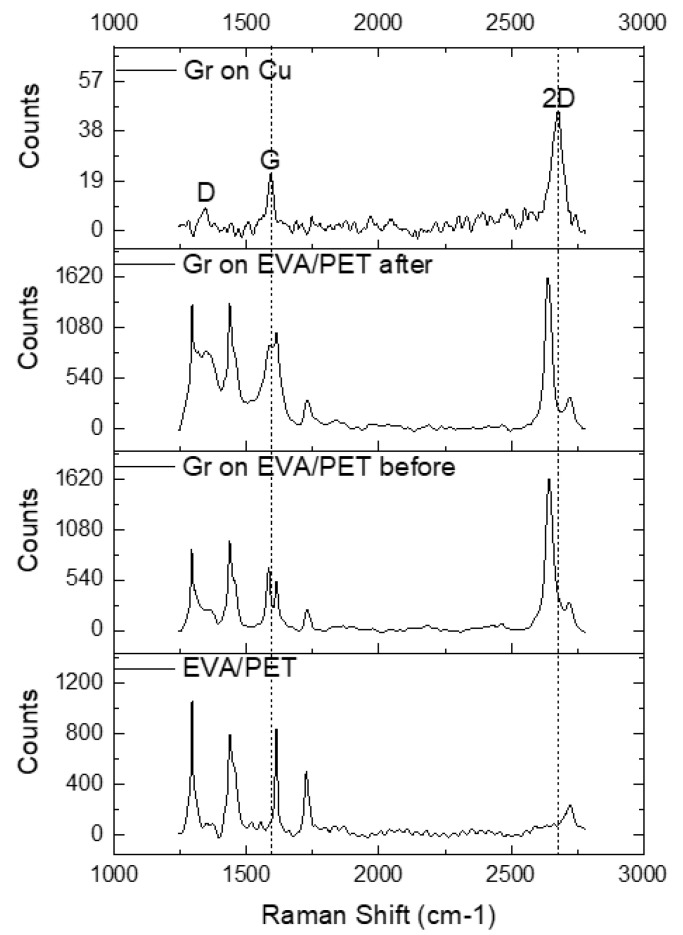
The Raman spectra of EVA/PET, graphene transferred to EVA/PET and CVD graphene on Cu.

**Figure 5:**
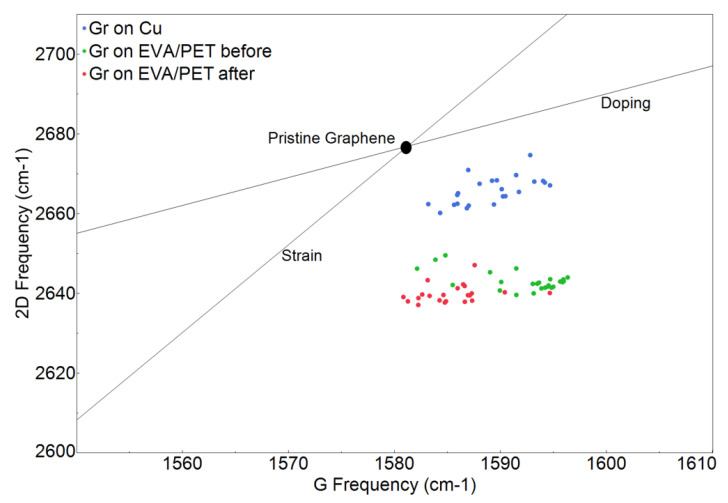
Frequency-correlation Raman map.

The Raman spectra of EVA/PET, graphene transferred to EVA/PET and CVD graphene on Cu are shown in Figure 4. A symmetric and sharp 2D peak for graphene on Cu indicates a presence of single layer graphene (signal predominately coming from monolayer region as seen in SEM). The high quality of CVD growth on Cu was also confirmed by a low intensity of the D peak. The transfer of graphene to EVA/PET was evident from the Raman spectrum, showing the characteristic G and 2D peaks. However, the D peak of graphene overlaps with one of the PET peaks and is hard to analyze. The frequency shifts of 2D and G peaks can be explained by the mechanical and chemical modification that graphene undergoes when transferred from Cu to EVA/PET. To investigate the spatial variation of the spectral features, multiple spectra were obtained by raster scanning the laser spot over the sample. Figure 5 shows the analyzed Raman-mapping results for the graphene on Cu and the graphene transferred to EVA/PET (before- and after the temperature treatment) extracted from 25 Raman spectra on each sample. The G and 2D band frequencies are sensitive to both strain and doping state of graphene. It was however reported by Lee et al. Ref. [27] that the fractional variation caused by strain and doping was very different. The 2D-vs.-G-frequency plot reveals the difference by using a simple vector model with separate unit vectors for the strain (the line with the slope of ∼2.2) and the doping (the line with the slope of ∼0.7). The strain line represents a prediction for a charge-neutral graphene under the uniaxial stress. The doping line, on the other hand, represents a varying density of charge carriers in graphene. We present our results in this plot to show the correlation map of the G-band-versus 2D-band frequency (see Figure 5).

The strain and doping lines cross at a point corresponding to a suspended exfoliated graphene with neither doping nor strain [27]. Almost all the spectra taken from graphene on Cu (blue dots) are clustered close to the region typical for pristine graphene, yet another indication of good quality of the CVD graphene. However, graphene transferred to EVA/PET undergoes both chemical- and mechanical modifications, as evident from the vertical shift in the frequency correlation plot. Since the shift in the 2D-peak frequency is more prominent than in the G-peak frequency, we conclude that the lamination process results in a considerable strain in graphene during the transfer from Cu to EVA/PET [12]. Indeed, the lamination involves both heat and pressure applied to the supporting polymer and graphene on copper. The coefficient of thermal expansion (CTE) of copper and EVA differ by an order of magnitude, $16.6\times 10^{-6}$ and $180\times 10^{-6}{\;}^{\circ }C^{-1}$ , respectively, while CTE of graphene is $-1\times 10^{-6}{\;}^{\circ }C^{-1}$ and continues to be negative until 420 ${}^{\circ }$ C [13]. The combination of the non-uniform pressure and difference in CET’s results in a tensile strain after the lamination process, which is evident from the frequency correlation map. We also studied the impact of strain and doping in graphene transferred to EVA/PET before (immediately after etching with HNO ${}_{3}$ ) and after the annealing. The spectral points represented by green and red dots in Figure 5 correspond to graphene samples measured before and after the temperature treatment, respectively. The graphene transferred to EVA/PET moreover retains its strain state after temperature treatment. This is validated by a smaller shift in the 2D frequency position of spectral points (red and green) on the frequency correlation plot (Figure 5). However, upon annealing, the majority of the spectral points (red dots in Figure 5) show comparatively larger shift in the G frequency. The spectral points corresponding to the annealed graphene (red dots) have the G frequency similar to that of the pristine graphene. On the other hand, the spectral points corresponding to a freshly etched graphene with HNO ${}_{3}$ have the G band shifted to higher frequency. Such a blue shift in the G band results from *p*-doping in graphene [27]. It has been reported that HNO ${}_{3}$ introduces *p*-type donors in graphene due to its strong oxidizing nature [15,16]. This doping is validated in Figure 5 by the presence of corresponding spectral points (green dots) in the high *p*-doping region of the map. However, due to the volatility of such dopants, they are removed upon annealing, resulting in a shift of spectral points in the opposite direction. These Raman findings are consistent with our Hall-effect measurements.

**Figure 6:**
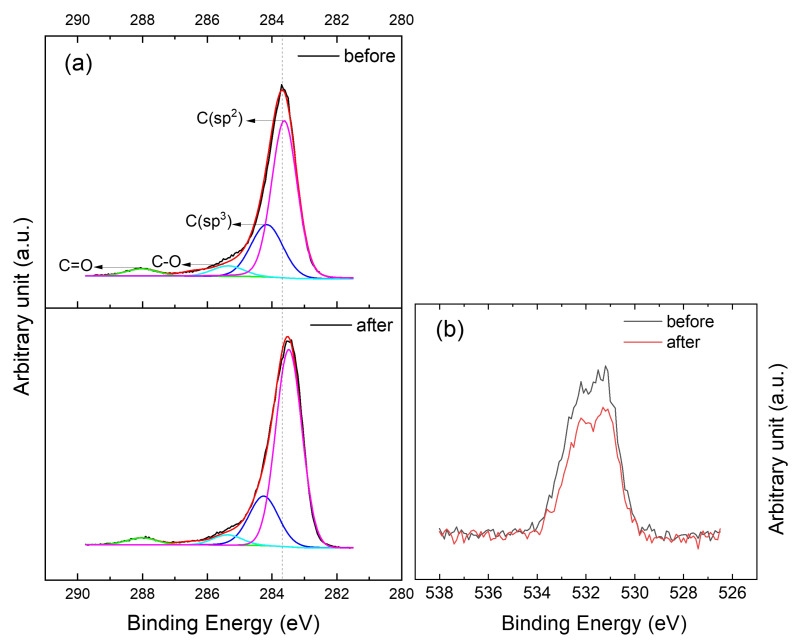
High resolution XPS narrow scanned spectra in (**a**) the C1s region (i) before and (ii) after annealing and (**b**) O1s region of the graphene on EVA/PET sample.

XPS measurements were carried out to quantify the chemical composition of our samples after etching and after annealing.The sample composition before annealing consists of 91.6 at.% of carbon and 8.4 at.% of oxygen and becomes 93.6 at.% of carbon and 6.4 at.% of oxygen afterwards (see Supplementary Information Figure S5). The introduction of oxygen functional groups by HNO ${}_{3}$ in graphene is confirmed by high resolution C1s and O1s spectra as shown in Figure 6. The C1s peak is deconvoluted into four main components by the fitting routine using 70% Gaussian-Lorentzian function after Shirley background removal. The four fitted deconvoluted peaks are centered at 283.5, 284.3, 285.4 and 288 eV, respectively. The peak at 283.5 eV is assigned to C(sp ${}^{2}$ ), while the peaks at 284.3, 285.4 and 288 eV are attributed to C(sp ${}^{3}$ ), C–O and C=O, respectively [2]. These peaks reveal the presence of various functional groups on the surface of CVD graphene transferred to EVA/PET. The area percentage of each group was estimated using the PHI Multipak™ software. It is worth mentioning that XPS data was recorded on CVD graphene transferred to EVA/PET. Therefore, it is quite difficult to eliminate the signal from oxygen of the underlying substrate. However, the 60 ${}^{\circ }$ C temperature treatment is unlikely to cause any change in the oxygen concentration of EVA/PET. Hence, all the changes observed after the heat treatment are most likely coming from the surface of graphene. Table 1 shows the area ratio of various functional groups before and after annealing. C(sp ${}^{2}$ ) and C(sp ${}^{3}$ ) are the dominant groups in which the proportion of each type is increasing and decreasing, respectively, upon annealing. Oxygen containing groups attached to the surface of graphene are often reported to be physisorbed [24]. Hence, their attachment and detachment can be reversible. This demonstrates that the HNO ${}_{3}$ treatment indeed introduces more functional groups on the surface of graphene, which eventually leads to *p*-doping. Concentration of the functional groups, however, is reduced upon annealing (Table 1). These XPS observations are in agreement with our controlled HNO ${}_{3}$ treatment (the inset in Figure 2b), where mobility increases every time the samples are annealed after being treated with HNO ${}_{3}$ . The functional groups at the surface of graphene tend to increase the doping and carrier scattering. This results in lower mobility of charge carriers, as observed during HNO ${}_{3}$ treatments. Upon annealing, the removal of these functional groups, validated by XPS measurements, reduces the carrier scattering and increases the carrier mobility. The oxygen groups are known for their electron affinity. Adsorption of these species on graphene, therefore, results in a higher binding energy position of the main peak of the C1s spectrum as shown in Figure 6a. Vice versa, upon removal of these groups after annealing, the C1s peak shifts to a lower energy. To further investigate the concentration of oxygen groups, O1s spectra were also analyzed (Figure 6b). From the O1s spectra shown in Figure 6b, it can be clearly inferred that the oxygen content, which is initially higher after treatment with HNO ${}_{3}$ , decreased upon annealing. The atomic percentage (at. %) of oxygen in graphene changed from 8.4 to 6.4 upon annealing. The higher amount of oxygen content in the graphene before annealing is attributed to the adsorption of oxygen groups on graphene introduced by its interaction with HNO ${}_{3}$ [28]. However, upon annealing, such molecules are desorbed from the surface of graphene. This reduction in oxygen and C(sp ${}^{3}$ ) content can in turn result in reduced scattering sites (and doping) explaining the increased Hall mobility observed in graphene on EVA/PET after annealing.

## 4. Conclusions

We demonstrate that HNO ${}_{3}$ etching of Cu from transferred CVD graphene on to EVA/PET introduces various functional oxygen groups. These functional groups result in *p*-doping of graphene. The increased dopant concentration causes charge carrier scattering and lower carrier mobility. However, due to the volatility of these functional groups, they easily desorb from the surface of graphene at 60 ${}^{\circ }$ C. This in turn reduces the charge carrier scattering, resulting in a significant increase in the carrier mobility of CVD graphene transferred to EVA/PET despite many double- and triple layer graphene patches seen in SEM. Hall effect measurement were conducted in order to quantify the carrier mobility of graphene samples before and after annealing. Raman and XPS measurements revealed the change in *p*-doping and oxygen functional groups respectively before and after annealing.

## Figures and Tables

**Table 1:** Area ratio of different chemical states assigned to C1s before and after annealing.

Peak	Area Ratio before	Area Ratio after
C(sp ${}^{2}$ )	63	71
C(sp ${}^{3}$ )	26	21
C–O	6	5
C=O	5	3

## Data Availability

The data presented in this study are available on request from the corresponding author.

## Supplementary Materials

The following supporting information can be downloaded at: https://www.mdpi.com/2079-4991/12/3/331/s1. Figure S1: Schematic of the Hall-effect system (left) and its practical realization (right). Figure S2: Photos of the adapter used in this work to quickly make contacts to a square-shaped piece of EVA/PET foil with CVD graphene. Figure S3: Contact shapes and placement (left) used in COMSOL simulations (right). The current is injected in between the lower pair of contacts while the voltage is calculated between the upper contacts. (a) Point contacts; (b) extended contacts ∼1 $\times 1\;{\mathrm{cm}}^{2}$ separated by the distance *s* (NB: measured from corner to corner). Figure S4: AFM images of graphene transferred to EVA/PET captured at two different positions on the sample. Figure S5: XPS survey scans of graphene transferred to EVA/PET by hot-press lamination before (Black) and after (Red) annealing at 60 ${}^{\circ }$ C.
