# Correction: Khan et al. High Mobility Graphene on EVA/PET. *Nanomaterials* 2022, *12*, 331

**DOI:** 10.3390/nano12111935

**Published:** 2022-06-06

**Authors:** Munis Khan, Kornelia Indykiewicz, Pui Lam Tam, August Yurgens

**Affiliations:** 1 Department of Microtechnology and Nanoscience, Chalmers University of Technology, 412 96 Göteborg, Sweden; kornelia.indykiewicz@chalmers.se (K.I.); avgust.yurgens@chalmers.se (A.Y.); 2 Faculty of Electronics, Photonics and Microsystems, Wrocław University of Science and Technology, Janiszewskiego 11/17, 50-372 Wrocław, Poland; 3 Department of Industrial and Materials Science, Chalmers University of Technology, 412 96 Göteborg, Sweden; eric.tam@chalmers.se

The authors wish to make following corrections in this paper [1]:

## Additional Affiliation

In the published study, there was an error regarding the affiliation for “Kornelia Indykiewicz”. In addition to affiliation 1, “Faculty of Electronics, Photonics and Microsystems, Wrocław University of Science and Technology, Janiszewskiego 11/17, 50-372 Wrocław, Poland” should also be acknowledged. 

## Additional Funding

In the original publication [1], the funder “Nordic Programme for Interdisciplinary Research”, “105121” to “Munis Khan and August Yurgens” was not included. The corrected Funding appears below.

**Funding:** This research has received funding from the European Union’s Horizon 2020 research and innovation programme under the Marie Skłodowska-Curie grant agreement No 955626 and Nordic Programme for Interdisciplinary Research, grant 105121. K.I. and A.Y. acknowledge support from the FLAG-ERA program, grant DeMeGRaS.

The authors apologize for any inconvenience caused and wish to state that the scientific conclusions are unaffected. This correction was approved by the Academic Editor. The original publication has also been updated.
