# Supplementary material for: High Mobility Graphene on EVA/PET
Source: Nanomaterials (Basel). 2022 Jan 21;12(3):331. doi: 10.3390/nano12030331 (PMC8840416; doi:10.3390/nano12030331)
Supplement: Supplementary file 1 [file nanomaterials-12-00331-s001.zip › nanomaterials-1532132-SM.pdf]

nanomaterials

MDPI

Supplementary Materials

High Mobility Graphene on EVA/PET

Munis Khan [1,*], Kornelia Indykiewicz [1], Pui Lam Tam[2] and August Yurgens [1]

[1] Department of Microtechnology and Nanoscience, Chalmers University of Technology, 412 96 Goteborg, Sweden; kornelia.indykiewicz@chalmers.se (K.I.); avgust.yurgens@chalmers.se (A.Y.)
[2] Department of Industrial and Materials Science, Chalmers University of Technology, 412 96 Goteborg, Sweden; eric.tam@chalmers.se
* Correspondence: munis@chalmers.se

Hall-effect mobility measurements in van der Pauw's contact configuration

The mobility of charge carriers in our samples have been measured by using a home-made setup using the rotated magnetic field (see Figure S1 for the schematic and photo of the system).[1] The samples were square-shaped, cut from 2″-large pieces of CVD graphene on EVA/PET foils and were contacted in four points close to the corners by using spring-loaded contact pins. In multiple measurements, a cushion of silver glue was made at each contact spot, to protect the graphene from being scratched away by metal pins (see Figure S2).

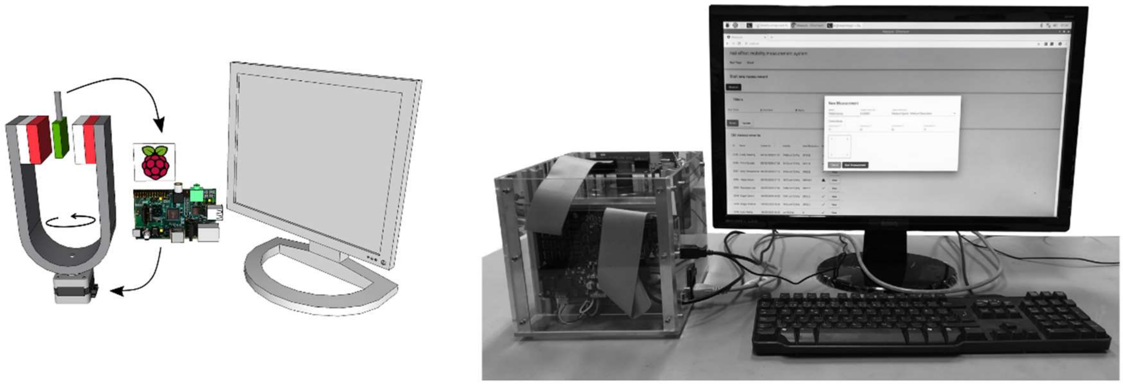

Figure S1. Schematic of the Hall-effect system (left) and its practical realization (right). The stepping motor, permanent magnets on a soft-iron core, Raspberry Pi single-board computer, the signal-conditioning- and contact-switching electronics, and a power supply are all assembled in a compact cubicle ~20 x 20 x 20 cm^3 large. The measurements are fully automated.

[1] Alma Blombäck, Anton Lööf, and Alexander Oxklint, *Design of Hall-effect measurement system*, Bachelor-degree thesis, Chalmers University of Technology, Gothenburg, Sweden, 2019 (in Swedish).

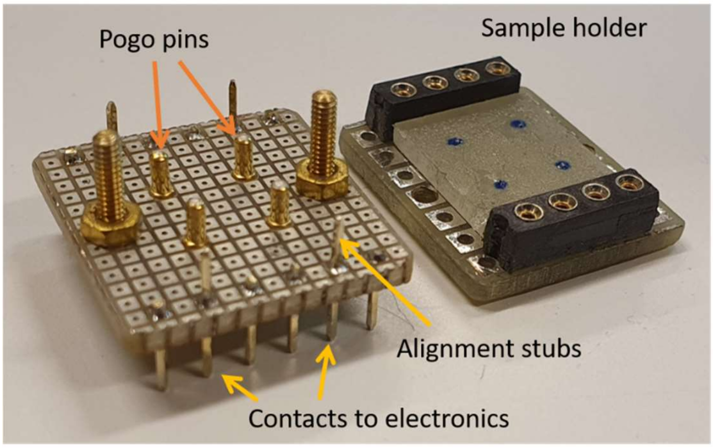

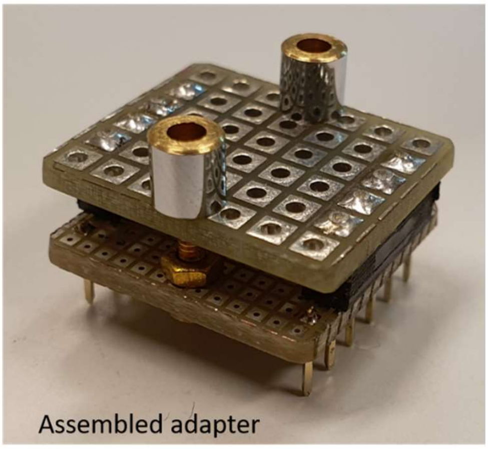

Figure S2. Photos of the adapter used in this work to quickly make contacts to a square-shaped piece of EVA/PET foil with CVD graphene.

The Hall-mobility-measurements theory for square samples is well known and is described in several papers.[2,3] Practically, the detailed instructions for such measurements are best presented at NIST (https://www.nist.gov/pml/nanoscale-device-characterization-division/popular-links/hall-effect) and Tektronix (https://www.tek.com/fr/document/whitepaper/hall-effect-measurements-materials-characterization). We follow these algorithms in our measurement setup.[1]

One concern might be related to the fact that the contacts are usually placed somewhat inside the sample area, i.e., not exactly at the sample perimeter, which is required for the original theory to be applicable.[4] While this has been thoroughly analyzed in e.g., Ref. S2, we show below simple COMSOL simulations, which clearly illustrate that the corresponding errors in, say, the sheet-resistance estimations can be easily kept below 10-15%.

[2] Daniel W. Koon, *Effect of contact size and placement, and of resistive inhomogeneities on van der Pauw measurement,* Rev. Sci. Instr. **60**, 271 (1989); doi: 10.1063/1.1140422

[3] S. H. N. Lima, D. R. McKenzie, and M. M. M. Bilek, *van der Pauw method for measuring resistivity of a plane sample with distant boundaries,* Rev. Sci. Instr. **80**, 075109 (2009); doi: 10.1063/1.3183503.

[4] L. J. van der Pauw, Philips Res. Rep. **13**, 1 (1958).

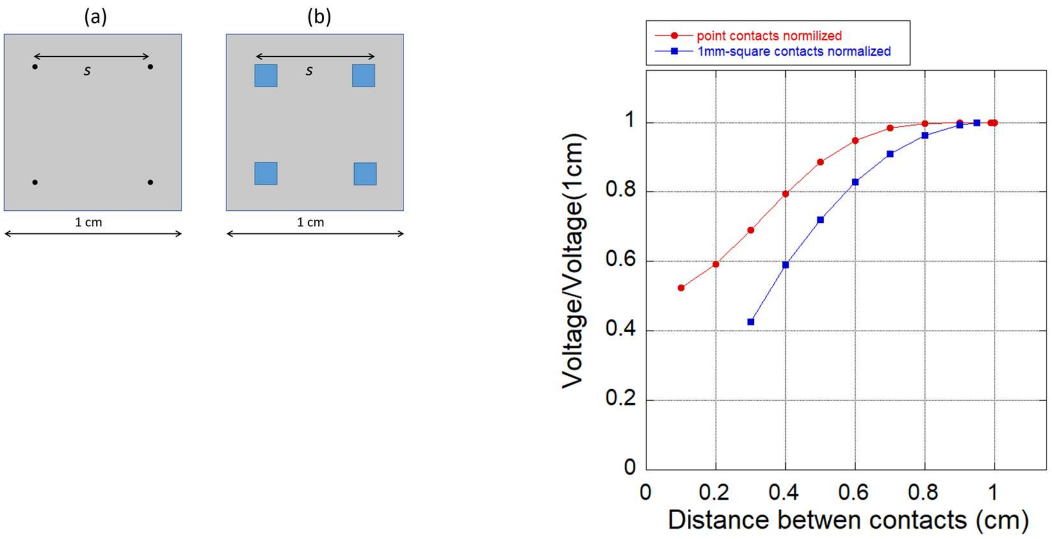

Figure S3. Contact shapes and placement (left) used in COMSOL simulations (right). The current is injected in between the lower pair of contacts while the voltage is calculated between the upper contacts. (**a**) Point contacts; (**b**) extended contacts 1 x 1 cm^2 separated by the distance *s* (NB: measured from corner to corner).

Atomic force microscopic height profile of graphene on EVA/PET

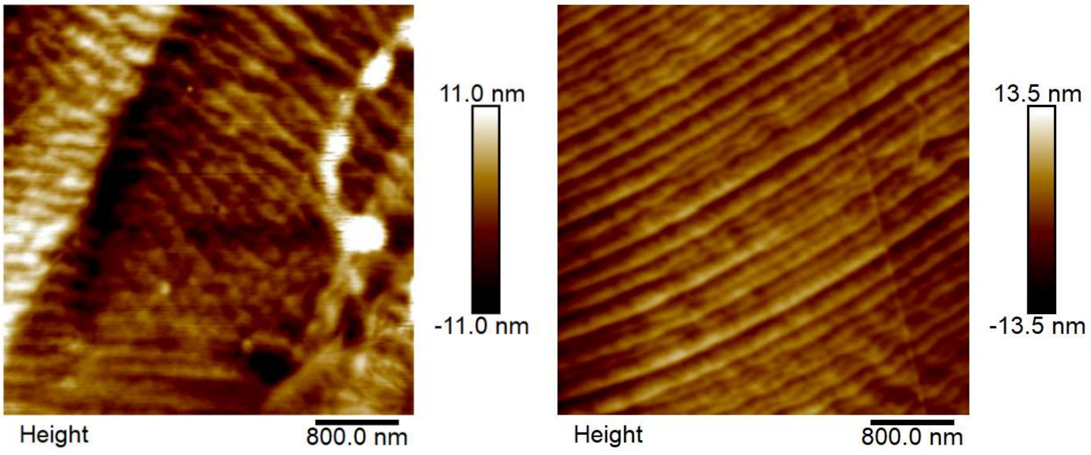

Figure S4. AFM images of graphene transferred to EVA/PET captured at two different positions on the sample.

XPS survey scans of graphene on EVA/PET

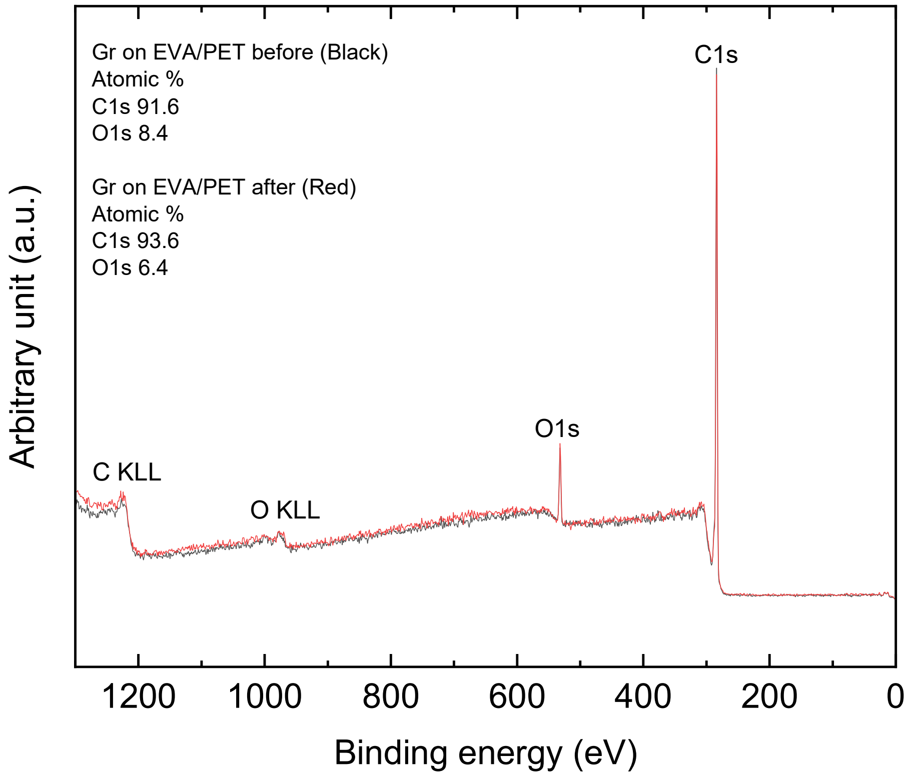

Figure S5. XPS survey scans of graphene transferred to EVA/PET by hot-press lamination before (Black) and after (Red) annealing at 60°C.
